# The Polo-Like Kinase 1 (PLK1) Inhibitor NMS-P937 Is Effective in a New Model of Disseminated Primary CD56^+^ Acute Monoblastic Leukaemia

**DOI:** 10.1371/journal.pone.0058424

**Published:** 2013-03-08

**Authors:** Alessia Casolaro, Josee Golay, Clara Albanese, Roberta Ceruti, Veronica Patton, Sabrina Cribioli, Alice Pezzoni, Marco Losa, Gemma Texido, Ursula Giussani, Francesco Marchesi, Nadia Amboldi, Barbara Valsasina, Silvia Bungaro, Gianni Cazzaniga, Alessandro Rambaldi, Martino Introna, Enrico Pesenti, Rachele Alzani

**Affiliations:** 1 Oncology, Nerviano Medical Sciences, Nerviano, Milano, Italy; 2 Laboratory of Cellular Therapy “G. Lanzani”, USC Haematology, Ospedali Riuniti, Bergamo, Italy; 3 Department of Oncology and Haematology, Istituto Clinico Humanitas, Rozzano, Milano, Italy; 4 Pathology, Fondazione Filarete, Milano, Italy; 5 Department of Medical Genetics, Ospedali Riuniti, Bergamo, Italy; 6 Pathology, Accelera S.r.l., Nerviano, Milano, Italy; 7 Tettamanti Research Center, Pediatric Clinic, University of Milano–Bicocca, Monza, Italy; European Institute of Oncology, Italy

## Abstract

CD56 is expressed in 15–20% of acute myeloid leukaemias (AML) and is associated with extramedullary diffusion, multidrug resistance and poor prognosis. We describe the establishment and characterisation of a novel disseminated model of AML (AML-NS8), generated by injection into mice of leukaemic blasts freshly isolated from a patient with an aggressive CD56^+^ monoblastic AML (M5a). The model reproduced typical manifestations of this leukaemia, including presence of extramedullary masses and central nervous system involvement, and the original phenotype, karyotype and genotype of leukaemic cells were retained *in vivo*. Recently Polo-Like Kinase 1 (PLK1) has emerged as a new candidate drug target in AML. We therefore tested our PLK1 inhibitor NMS-P937 in this model either in the engraftment or in the established disease settings. Both schedules showed good efficacy compared to standard therapies, with a significant increase in median survival time (MST) expecially in the established disease setting (MST = 28, 36, 62 days for vehicle, cytarabine and NMS-P937, respectively). Importantly, we could also demonstrate that NMS-P937 induced specific biomarker modulation in extramedullary tissues. This new *in vivo* model of CD56^+^ AML that recapitulates the human tumour lends support for the therapeutic use of PLK1 inhibitors in AML.

## Introduction

AML is a heterogeneous disease that accounts for about 30% of all adult leukaemias. The heterogeneity of AML was first evidenced by morphological and immunophenotypic analyses of the neoplastic cells. More recently an extensive genetic heterogeneity has also been identified, with a wide range of genetic alterations detected, such as chromosomal imbalances (42% of patients), recurrent chromosomal translocation/inversions/deletions involving different chromosomes (15%) and point mutations of specific genes, including *FLT3, CEBPA, NPM1, DNMT3*, to cite only the most frequent events [1]–[3]. Several of the genetic alterations or phenotypic markers have a prognostic impact on therapy and/or can be novel drug targets [4]–[7].

CD56 (NCAM) expression is one of the prognostic markers in AML. It is heterogeneously expressed in different haematopoietic neoplasms, including AML, lymphomas and myelomas [8]. CD56 expression has usually been associated with poor prognosis in different contexts and with extramedullary presentation of AML, in particular meningeal involvement [9]–[11].

Conventional treatment for AML patients is based on a combination of anthracyclines and cytosine arabinoside (ara-C). However the heterogeneity of AML leads to response rates varying from 30% to 90%, evidencing the need for new treatment options, especially for poor prognosis AML subtypes [1], [12]. PLK1 is a serine/threonine (ser/thr) kinase involved in cell cycle regulation at the G2/M transition and it is a direct target of the oncogenic kinase Aurora A [13]. It is overexpressed in a variety of malignant solid tumours and the development of inhibitors targeting this mitotic kinase has already reached clinical trials with encouraging, even if preliminary, evidence of antitumour activity [14]. Recently, PLK1 has started to be considered a possible therapeutic target also in myeloid leukemias, since it is overexpressed in the majority of AML cells and these cells respond to PLK1 inhibitors *in vitro* and *in vivo* [15].

New orthotopic xenograft models using leukaemic cells directly derived from patients have been developed and are an important tool for the preclinical evaluation of new drugs since they are likely to better represent the human disease [16]–[22]. In this study, we report the successful establishment and extensive characterisation of a novel model of aggressive disseminated AML, generated by injecting into mice primary CD56^+^ leukaemic cells (AML-NS8) obtained from an AML-M5a patient bearing trisomy 8 and 6. The tumour is aggressive *in vivo* in SCID mice, and in large part recapitulates the human disease with both medullary and extramedullary leukaemic infiltration. Furthermore the AML-NS8 cells responded favourably to our Polo-Like Kinase 1 (PLK1) inhibitor NMS-P937 compared to standard therapy, both *in vitro* and *in vivo*. These data suggest that PLK1 is a promising drugable target for AML therapy.

## Materials and Methods

### Patient and Cells

The adult patient AML-NS8 presented in hospital with a hypercellular bone marrow (BM) infiltrated by >90% with CD34^−^, HLA-DR^+^, CD33^+^, CD56^+^, CD7^−^, CD19^−^, α-napthyl acetate esterase (ANAE) positive monoblast like cells. Cytogenetic analysis (Q-banding) of the BM showed presence of 2 AML clones, respectively with trisomy 8 e double trisomy 8 and 6 (7 and 15 metaphases out of 22, respectively). PCR analysis of BM sample showed negativity for *AML1-ETO* and *CBFβ/MYH11* rearrangements, *MLL* partial tandem duplication, *NPM1*, *CEBPA* and *FLT3-ITD and TDK* mutations. The peripheral blood count was above 200000 wbc/µl. He was diagnosed with AML M5a according to FAB classification. The patient was enrolled in the NILG AML 01/00 clinical protocol “Risk-oriented therapeutic strategy for adult acute myelogenous leukaemia” (Clinicaltrials.gov n° NCT00400673). Leukapheresis was performed and a first dose of cytarabine infused to reduce the tumour burden. He died few weeks after appearance of first symptoms. Leukaemic cells were obtained from the leukapheresis after written informed consent and approval by the local institutional ethics committee (Comitato di Bioetica, Ospedali Riuniti, Bergamo). Mononuclear cells were isolated by Ficoll-Hypaque gradient centrifugation and aliquots frozen in 10% dimethyl sulphoxide.

### Drugs

NMS-P937 is a previously described PLK1 inhibitor [23]. Cytarabine (ara-C) was from Hospira (Lake Forest, IL, USA) and doxorubicin from Bedford Laboratories (Bedford, OH, USA).

### *In vivo* Expansion of AML-NS8 Cells

All procedures adopted for housing and handling of animals were in strict compliance with European and Italian Guidelines for Laboratory Animal Welfare. The protocol was approved by the Ethics of Animal Experiments Committee of Nerviano Medical Sciences. All efforts were made to minimize suffering. 50–100×10^6^ thawed AML-NS8 cells were injected intraperitoneally (ip) into irradiated NOD/SCID mice (Charles River Laboratories-Calco, Italy), as previously described [17], [18]. Animals were sacrificed when ascitic fluid was observed and leukaemic cells were collected from the abdominal cavity. Recovered AML-NS8 cells were further expanded in groups of 5–10 SCID mice (Charles River Laboratories) by serial ip passages using 10–20×10^6^cells/mouse. After 5 *in vivo* passages, the phenotype and genotype of collected and pooled cells were verified by flow cytometry, cytogenetic and SNP arrays analysis. These cells were frozen in aliquots for further studies.

### Disseminated Primary Leukaemia Model and Treatment Protocol

Unless otherwise indicated 5×10^6^ pooled *in vivo* expanded AML-NS8 cells (from 5^th^ passage) were transplanted into 5 weeks old SCID mice by tail-vein injection (iv). Animals were monitored for the insurgence of leukaemic signs, sacrificed when moribund and autopsied. For histopathology column, femour, sternum, whole skull, spleen, stomach, gut, liver, kidney, skin and lung tissues were collected. For flow cytometry, blood was taken by retro-orbital bleeds, BM was obtained by flushing it from femurs with PBS and spleen was mechanically dissected.

For *in vivo* experiments, in the engraftment setting (preemptive protocol) treatments started on day 4 after AML-NS8 iv injection. Mice were randomly assigned to one of the following groups: vehicle ip, cytarabine (75 mg/kg ip per day over 5 days for 4 cycles with 7 day rest), doxorubicin (3 mg/kg iv every 7 days for 3 cycles) or NMS-P937 (120 mg/kg os per day over 2 days for 4 cycles with a 10 day rest). Furthermore, in the established disease setting (therapeutic protocol), treatments started on day 20 after AML-NS8 injection when leukaemic dissemination was evaluable and mice were randomly divided into the following groups: vehicle ip, cytarabine (75 mg/kg ip per day over 5 days with 5 day rest, continued until mice were moribund), or NMS-P937 (60 mg/kg bid os per day over 2 days with a 5 day rest, continued until mice were moribund). Animals were monitored for clinical signs of disease and time of death recorded. All animals were autopsied.

### Histology and Immunohistochemistry

Organs collected were fixed in 10% buffered formalin for 24 hours and 5% formic acid was added to decalcify bone structures. Tissues were prepared, stained with haematoxylin and eosin (H&E) and processed for immunohistochemistry as previously described [14], [24]. Briefly, slides were heat unmasked using low (Vector Laboratories, Burlingame, CA, USA) or high (Dako, Glostrup, Denmark) pH solutions and incubated with primary antibodies anti-human HLA,A,B,C (MBL, Woburn, MA, USA), anti-human phospho-H3 (Upstate, Charlottesville, VA,USA), phospho-NPM1 phospho-TCTP (Cell Signaling, Danvers, MA, USA) and active Caspase 3 (Cell Signaling, Danvers, MA, USA). Envision+ System HRP anti-mouse and rabbit (Dako, Glostrup, Denmark) were used as secondary antibodies.

### *In vitro* Expansion

Pooled *in vivo* expanded AML-NS8 cells from passage 5 were cultured at 0.5–1×10^6^/ml in RPMI-1640 medium (Invitrogen, Carlsbad, CA, USA), supplemented with 20% foetal calf serum (Euroclone, Wetherby, West Yorkshire,UK), 1 ng/ml recombinant human granulocyte-macrophage colony-stimulating factor (rhGM-CSF) and 10 ng/ml interleukin-3 (rhIL-3)(both from Sigma-Aldrich, Gillingham, UK). To calculate doubling time, cells were seeded at 50×10^3^cells/ml in duplicates in complete medium and counted daily in a Coulter Counter (Coulter, Brea, CA, USA) for 8 days.

### *In vitro* Assays

AML-NS8 cells (2000 cells/well) were seeded in 384 well-plates in complete medium and incubated at 37°C and 5% CO_2_. After 24 hours increasing concentrations of doxorubicin (range 1 µM - 0,004 µM), cytarabine or NMS-P937 (range 10 µM - 0,001 µM) were added and incubation continued for further 72 hours. Cells were then processed by CellTiter-Glo assay (Promega, Madison, WI, USA) following the manufacturer’s instructions. Inhibitory activity was evaluated using the Symyx Assay Explorer (Symyx Technologies Inc., Santa Clara, CA, USA) program. The concentration resulting in 50% inhibition (IC_50_) was calculated using the sigmoidal interpolation curve.

For *in vitro* mechanism of action studies, AML-NS8 cells were seeded at 1.5×10^6^/ml and treated with 0.2 µM NMS-P937 for 24 hours or 0.75 ng/ml Nocodazole for 16 hours. One part of cells were then lysed for western blotting and the other fixed in ethanol 70% and stained with Alexafluor 488 conjugated anti-phospho-Histone H3 antibody (Cell Signaling, Danvers, MA, USA) and propidium iodide (Sigma Aldrich, Gillingham, UK) for FACS analysis.

### Flow Cytometry

For standard immunophenotyping, cells in suspension were stained with CD3-PE, CD19-FITC, CD33-PE, HLA-DR-PE, CD10-FITC, CD123-PE, CD25-PE, CD11b-PE, CD18-FITC, CD56-PE (all from BD Biosciences, Franklin Lakes, NJ, USA), CD52-PE (Caltag Laboratories, Burlingame, CA), CD117-PE (Immunotech, Mississauga, ON, Canada), and analysed by FACSCalibur (BD Biosciences, Franklin Lakes, NJ, USA).

### Western Blotting

Cells extracts and immunoblotting were performed as previously described [14] and staining was done using the following primary antibodies against: PLK1 (Zymed, San Francisco, CA, USA), NPM1 and pThr199 NPM1 (both from Cell Signaling, Danvers, MA, USA), Histone H3 (Abcam, Cambridge, UK), pSer10 Histone H3 (Upstate, Charlottesville, VA,USA). Secondary anti-mouse-HRP or anti-rabbit-HRP conjugates were from Thermo Scientific (Waltham, MA, USA) and ECL detection reagent from Amersham (GE Healthcare Ltd, Amersham, UK).

### Single Nucleotide Polymorphisms (SNP) Arrays Analysis

Diagnostic, *in vivo* (passage 5) and *in vitro* expanded AML-NS8 cells (passage 15) were genotyped using Affymetrix® Cytogenetics Whole Genome 2.7 M Array (Affymetrix®, Santa Clara, CA, USA), as previously described [25].

## Results

### *In vivo* and *in vitro* Expansion of AML-NS8 Cells

Since expression of CD56 (NCAM) has been observed in a significant proportion of cases of AML, ALL, MM and B-NHL and is associated with extramedullary involvement and poor prognosis, we have selected one case of aggressive CD56^+^ AML for *in vivo* inoculation in mice. In this study AML-NS8 cells were directly derived from a patient with an aggressive CD56^+^ AML (whose characteristics are described in Materials and Methods) and were injected intraperitoneally (ip) into NOD/SCID mice. These cells engrafted well and expanded within the abdominal cavity, leading after a few weeks to the production of both ascites and solid tumour masses in this body location. Repeated passages of cells ip into SCID mice allowed their expansion to generate a large pool of leukaemic cells. Their phenotype was analyzed using a panel of 16 antibodies directed against cell lineage markers and cytokine receptors and it was identical to that of the blasts at diagnosis, including lack of expression of CD34, CD117, CD25, CD3, CD7 and CD19 and clear expression of the myeloid markers CD13, CD33, CD11b, CD11c, CD18, HLA-DR, CD10, CD123 (IL-3R), CD52 and notably of CD56 (NCAM). Furthermore, the expanded cells stained positive for fluoride sensitive ANAE by cytochemical staining, like the diagnostic sample (Table 1).

**Table 1:** Phenotype of AML-NS8 cells, at diagnosis, expanded ip in mice or expanded *in vitro* with rh-IL3 and rh-GM-CSF.

		Phenotype*		
Antigen	Antigen function	Leukapheresis (diagnosis)	*In-vivo* expanded cells	*In vitro* cultured cells
CD3	TCR	neg	neg	neg
CD7	T cell marker	neg	ND	ND
CD19	Costimulatory, pan-B	neg	neg	neg
CD34	HSC marker	neg	neg	neg
CD13	alanine aminopeptidase	++	+	++
CD33P	SIGLEC lectin	++	++	++
HLA-DR	Histocompatibility	++	+++	++
CD10	Metalloproteinase	+	+	+
CD123	IL3R	+	ND	+
CD25 P	IL2R	neg	neg	neg
CD117	SCF-R,c-kit	neg	neg	neg
CD11b	heterodimer: MAC1, CR3	+++	++	++
CD18	heterodimer: MAC1, CR3	+++	++	++
CD11c	CR4	++	ND	+
CD52	GPI-linked,alemtuzumab target	+++	++	++
CD56	NCAM	++	++	+
ANAE**	a-napthyl acetate esterase	+++	+++	+++

* Neg: <10% staining; +: <80% staining or MFI<100; ++: 80–100% staining and MFI 100–400; +++: 80–100% staining and MFI>401 by flow cytometry. ND: not done. Immunophenotypes were performed three times.

** Cytochemical staining for fluoride sensitive ANAE.

**Figure 1:**
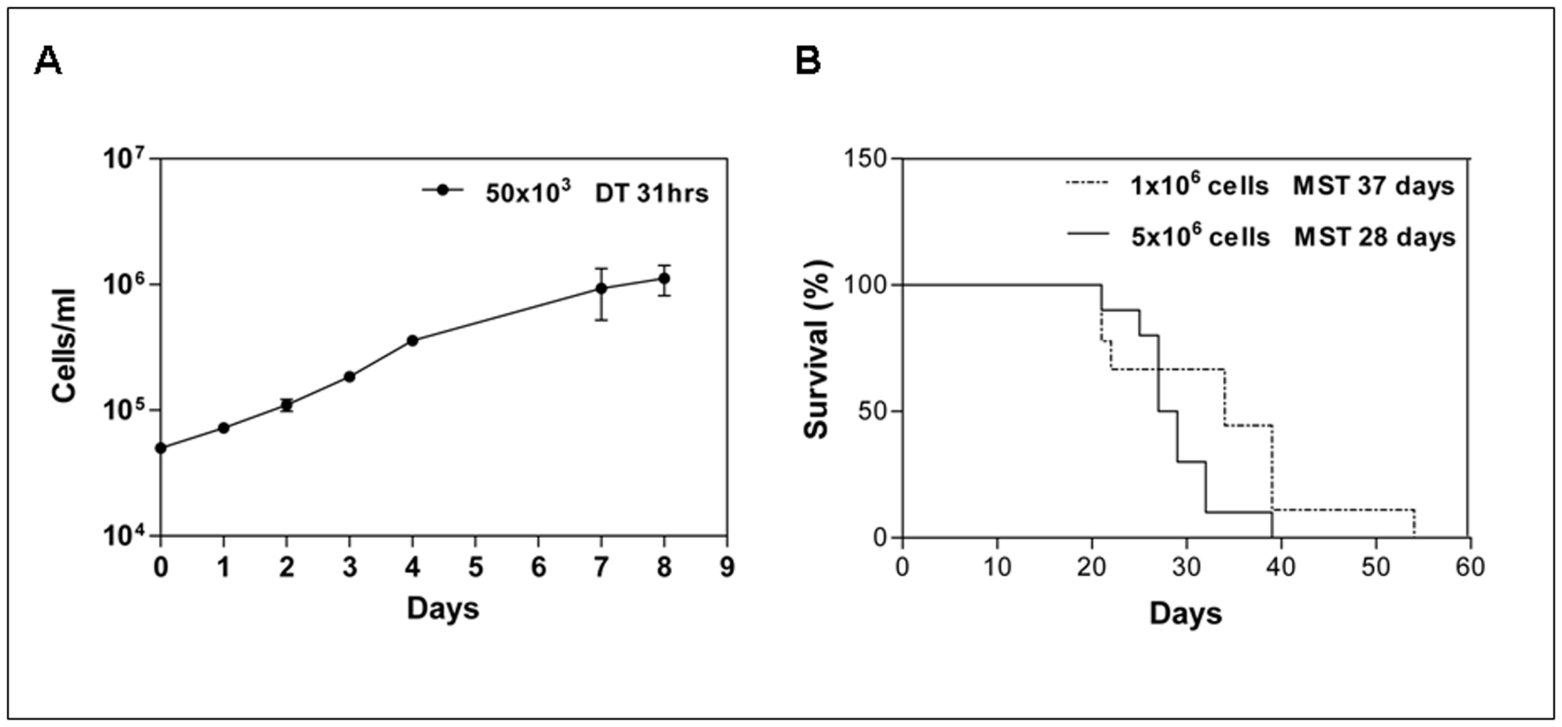
*In vitro* and *in vivo* growth of AML-NS8 cells. (**A**) Neoplastic cells were plated *in vitro* at 50×10^3^ cells/ml in presence of rhIL-3 and rhGM-CSF. Cells were counted at the indicated times to determine the doubling time (DT = 31hours). The data are representative of 3 experiments. (**B**) On day 0, groups of 10 SCID mice were inoculated iv with either 1×10^6^ (dotted line) or 5×10^6^ (continuous line) *in vivo* expanded AML-NS8 cells and survival time recorded. The median survival time (MST) was 37 and 28 days respectively.

In order to establish also an *in vitro* growing cell line for preliminary drug testing and mechanistic studies, AML-NS8 cells were put in culture in the presence of growth factors. In these conditions cells were able to expand *in vitro*, with a doubling time of 31 hours (Figure 1A) and could be cultured for at least 3 months, preserving their phenotypical identity with sample at diagnosis (Table 1).

The karyotype of the *in vivo* and *in vitro* expanded cells was also compared to that of the leukapheresis and BM samples, both taken at diagnosis. By standard Q-banding, the leukapheresis sample showed the double trisomy of chromosomes 6 and 8 and this was observed in 28 out of 31 metaphases. The other 3 metaphases were apparently normal, suggesting that they represented contaminating normal cells present in peripheral blood (Table 2). These data suggest that in the periphery, only the clone with double trisomy was present, in contrast to BM, where an additional clone with only trisomy 8 was also detected and represented about 32% of analysed metaphases (Table 2 and Figure S1A). The double trisomy 6/8 was shown in all 28 metaphases from pooled *in vivo* expanded cells at passage 5, as expected since they derived from the leukapheresis sample (Table 2 and Figure S1B) and this data was also confirmed by FISH using chromosome 6 and 8 probes (Figure S1C). The *in vitro* expanded cells, as expected, showed in all metaphases the double chromosome 6/8 trisomy, but also a small deletion on the short arm of chromosome 10 difficult to clearly identify (Table 2). No other cytogenetic abnormalities [*NPM1*, *CEBPA* and *FLT3* (*ITD and TKD*) mutations, *MLL* partial tandem duplication, *AML1/ETO* and *CBFβ/MYH11* translocations] were detected in the diagnostic, *in vivo* and *in vitro* expanded cells by PCR analysis (data not shown).

**Table 2:** Karyotype and Genomic aberrations of AML-NS8 from different sources.

		Cytogenetics byQ-banding		Genomic aberrations bySNP arrays (Loci/size in kbp)									
Sample	Origin	N° metaphasesanalysed	Karyotype(metaphases %, M^a^)	Trisomy	FocalLosses							FocalGains	
				Chr 6, 8	3q26.32(27)	9p21.3(116)	10p12.31(415)	11p14.1(127)	11p11.2(143)	11q23.3(779)	17p13.1(62)	13q21.31-q34(50179)	14q 21.2(423)
Diagnosis	BM	22	48,XY+6,+8 (68%–15 M); 47,XY,+8 (32%–7 M)	ND^b^	ND	ND	ND	ND	ND	ND	ND	ND	ND
Diagnosis	Leukapheresis	31	48,XY,+6,+8 (90%–28 M); 46,XY (10%–3 M)	x^c^	x	x	x	x	absent	x	x	absent	x
*In vivo* expandedp5	Ascitic fluid	28	48,XY,+6,+8(100%–28 M)	x	x	x	x	x	absent	x	x	absent	x
*In vitro* expanded p15	Culture	30	48,XY,+6,+8 del(10)(p?13pter)(100%–30 M)	x	x	x	larger	x	x	larger	x	x	x

a M: metaphases.

b ND: not done.

c x: presence of aberration.

In order to assess in more details the genetic stability of AML-NS8 cells, whole genome SNP arrays analysis was performed on genomic DNA from the leukapheresis sample obtained at diagnosis, as well as from the *in vivo* and *in vitro* expanded cells. The genomic copy number (CN) profile obtained by SNP arrays on the diagnostic sample showed a number of deletions on chromosome loci 3q26, 9p21, 10p12, 11p14, 11q23 and 17p13, as well as a gain at the 14q21 locus, as detailed in Table 2. These deletions were too small to be detected by standard Q-banding. Parallel analysis of the genomic profiles of the *in vivo* expanded cells revealed that they were genetically stable with respect to the diagnostic leukapheresis sample, since they showed identical number and sizes of deletions and gains of genetic material (Table 2). In contrast, *in vitro* expansion of the cells induced the appearance of one *ex novo* genetic alteration on chromosome 11 and two larger deletions with respect to the leukapheresis sample, but at similar chromosomal positions (Table 2). The larger loss on chromosome 10p was also detected by Q-banding (Table 2). The considerable gain on chromosome 13 and the larger deletion on chromosome 11q23.3 were confirmed by FISH analysis using probes specific for these chromosomes (LAMP1, D13S319 and ATM, respectively). Thus FISH revealed that the gain of the long arm of chromosome 13 was joined to the deleted long arm of chromosome 11, such that this anomaly was not readily detectable by standard Q-banding (data not shown). Other anomalies, as for the diagnostic clone, were too small to be visible by Q-banding.

It is worth noting that all losses detected by SNP arrays were monoallelic, except that at locus 9p21 which was biallelic. This loss included a 116 kbp region that contained the tumour suppressor gene *CDKN2A* (p16^INK4A^) and *MTAP* (methylthioadenosine phosphorylase). This biallelic loss was present on all samples (Table 2).

These data demonstrate that *in vitro* growth of leukaemic cells induces genetic alterations which are not initially present or detectable at diagnosis. In contrast *in vivo* expansion of the cells preserves a more stable genotype.

### Characterisation of a Disseminated Model of AML-NS8 Leukaemia

In order to generate a model of disseminated leukaemia, AML-NS8 cells obtained from intraperitoneal passage 5 were inoculated by iv route in SCID mice. Two different AML-NS8 cell concentrations (1×10^6^ and 5×10^6^/mouse) were tested. As shown in Figure 1B, these two injections led to a median survival time (MST) of 37 and 28 days, respectively. Injection of 5×10^6^ cells was chosen for further experiments of disseminated leukaemia, since this schedule gave a reproducible MST of 29 days ±1 (calculated on 8 independent experiments).

**Figure 2:**
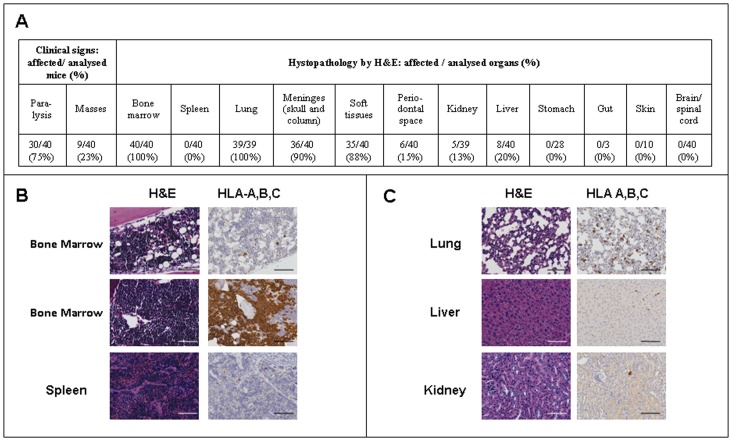
Histological and immunohistochemical analysis of different tissues. SCID mice were inoculated iv with 5×10^6^ AML-NS8 cells, monitored for clinical signs and sacrificed upon manifestations of terminal disease. Different tissues were collected and fixed for histology and immunohistochemistry. (**A**) Clinical signs and histopathological characterisation of AML-NS8 *in vivo* disseminated model. (**B, C**) Representative pictures (Axio Scope Zeiss, magnification×200) of the indicated tissues stained with H&E (left panels) or anti-human HLA-A,B,C (right panels). Black/white bar, 100 µm.

With the view to perform a complete analysis of the organs involved and of the localisation of tumour cells in the AML-NS8 disseminated model, 40 mice coming from 4 different experiments were monitored and euthanised when they showed manifestations of terminal disease. Clinical signs observed included hind limb paralysis in 75% of animals, presence of visible masses on the body surface in 23% of the cases (Figure 2A) and respiratory failure in the majority of the animals. At autopsy macroscopic analysis revealed that masses were always localised on the surface of bones, mainly of limbs, column and skull.

As shown in Figure 2A, the histopathological evaluation of collected organs showed the presence of neoplastic cells in 100% of marrow samples in femur, column or skull bones. In other haematopoietic organs, such as spleen and the few evaluable lymph nodes (data not shown), no neoplastic infiltration was detected by H&E. Several non haematological organs were also affected, since 100% of animals had neoplastic cells in the lungs and about 90% in meninges and soft tissues. In contrast only 13–20% of cases analysed had the kidney and liver infiltrated, while in stomach, gut, skin, brain and spinal cord neoplastic cells were never observed. Finally, invasion within the periodontal space of mandibular or maxillary bones was also detected.

In the organs where histopathology did not clearly allow to detect leukaemic infiltration, immunohistochemistry staining for human HLA-A,B,C showed the variable extent of infiltration of the different femoral bone marrow samples as well as the presence of only scattered neoplastic cells, single or in small groups, in spleen (Figure 2B) and also in lung, liver and kidney (Figure 2C). Flow cytometry results, performed on cellular suspensions stained with the same antibody, confirmed the variable infiltration in the femoral marrow (1–94%; median 24%), the low dissemination in the spleen (0–4%; median 1%) and showed neoplastic cells in all blood samples analysed (1–29%, median 6%).

The detailed analysis of macroscopic masses, exclusively composed of tumour cells as histopathologically revealed, showed that they infiltrated muscles and always were associated/adjacent to bone periosteum. The marrow contained in these bones was often replaced completely by tumoural cells and bone remodeling/resorption processes were evident (Figure S2A). Similarly in the skull, infiltration of the meninges was a prominent finding (Figure S2B), confirmed by preliminary data obtained by magnetic resonance imaging (MRI, Figure S2C).

We conclude that the disseminated AML-NS8 model recapitulates several clinical characteristics of extramedullary AML, with the involvement of both haematopoietic and non-haematopoietic organs, most prominently meninges, muscle and lung as well as the masses appearance.

### *In vitro* and *in vivo* Activity of PLK1 Inhibitor NMS-P937 on AML-NS8 Cells in Comparison with Standard Drugs

**Figure 3:**
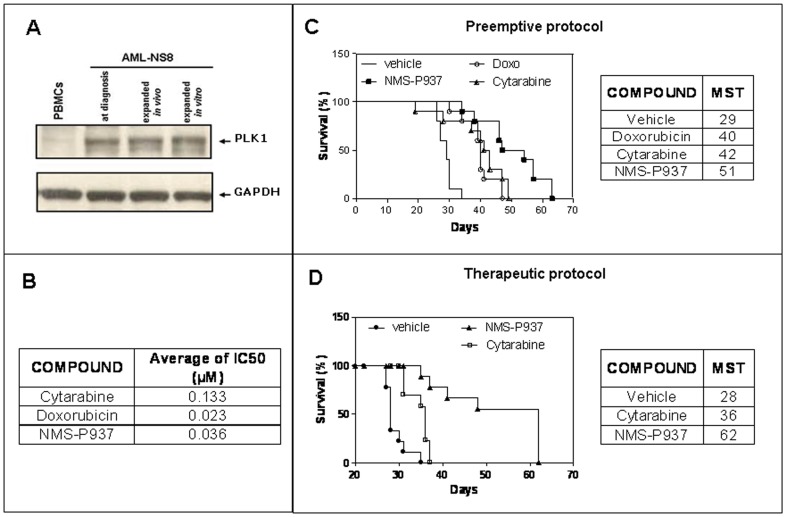
PLK1 expression in AML-NS8 cells and activity of PLK1 inhibitor NMS-937 *in vitro* and *in vivo*. (**A**) Western blot analysis. Leukaemic cells at diagnosis, expanded *in vivo* or *in vitro* and PBMCs from healthy volunteers were lysed and PLK1 expression analysed by Western blot. GAPDH protein expression was used as loading control. (**B**) Cytotoxicity assay. AML-NS8 *in vitro* expanded cells were incubated in presence or absence of increasing concentrations of cytarabine, doxorubicin and NMS-P937 for 72 hours and IC_50_ determined for each drug. (**C**) *In vivo* efficacy: preemptive protocol. Groups of 10 mice were inoculated iv with 5×10^6^ AML-NS8 cells and treated with vehicle, cytarabine at 75 mg/kg ip per day over 5 days for 4 cycles with a 7 day rest, doxorubicin at 3 mg/kg iv every 7 days for 3 cycles and NMS-P937 per os at 120 mg/kg per day over 2 days, for 4 cycles with a 10 day rest and survival recorded. The Kaplan-Meier plot and MST are shown. Statistical analysis using the Wilcoxon test showed that all drug treatments were statistically different from vehicle alone (p<0.01 for cytarabine and p<0.001 for NMS-P937 and doxorubicin). NMS-P937 was also statistically different from the other drugs with p<0.05. Data are representative of 2 independent experiments. (**D**) *In vivo* efficacy: therapeutic protocol. Groups of 10 mice were inoculated iv with 5×10^6^ AML-NS8 cells and treatments started at day 20, when a clear leukaemic dissemination was visible. Mice were treated with vehicle, cytarabine at 75 mg/kg ip per day over 5 days with a 5 day rest continuously until mice were moribund, and NMS-P937 per os at 60 mg/kg bid per day over 2 days with 5 day rest continuously until mice were moribund and survival recorded. The Kaplan-Meier plot and median survival times (MST) are shown. Statistical analysis using the Wilcoxon test showed that all drug treatments were statistically different from vehicle alone (p<0.0001 for each compound). NMS-P937 was also statistically different from cytarabine with p = 0.001.

The conventional induction and consolidation therapy for AML is a combination of cytarabine and anthracycline, but new drugs are required for aggressive and resistant forms of the disease. PLK1 has been demonstrated to be overexpressed in several AML patient samples and is a candidate target protein for therapy [15], [26], [27]. We therefore evaluated PLK1 expression in the diagnostic, *in vivo* and *in vitro* expanded AML-NS8 cells by Western blot analysis. As shown in Figure 3A, PLK1 was expressed in all samples, but not in normal peripheral blood mononuclear cells (PBMC).

Given the expression of PLK1 by AML-NS8 cells, we used these cells *in vitro* as target for the cytotoxic activity of our proprietary PLK1 inhibitor NMS-P937 [14], [23], in comparison with cytarabine and doxorubicin. The *in vitro* efficacy of the compounds, expressed as IC_50_ calculated after 72 h treatment, was 133 nM, 23 nM and 36 nM for cytarabine, doxorubicin and NMS-P937, respectively (Figure 3B). These data suggested an overall favourable activity of the PLK1 inhibitor *in vitro* against AML-NS8 cells.

The same drugs were then tested *in vivo* in the AML-NS8 disseminated model following a preemptive protocol as described in Materials and Methods. All compounds had activity compared to vehicle (MST of 29, 40, 42 and 51 days for vehicle, doxorubicin, cytarabine, and NMS-P937, respectively) (Figure 3C). Importantly, PLK1 inhibitor treatment produced a significant (p<0.05) increase of MST (51 days) compared to standard therapies (40–42 days) (Figure 3C). Similar results were obtained in 2 independent experiments.

On the basis of the significant effect of NMS-P937 in the preemptive protocol, we investigated the efficacy of the compound in an established AML-NS8 disease in comparison with cytarabine. In this experiment, treatments (as specified in Materials and Methods) started at day 20 after tumoural cells injection, since in previous time course experiments a clear dissemination was evaluable from the third week post engraftment (data not shown).

As shown in Fig 3D both compounds significantly prolonged survival compared to vehicle (MST = 28, 36, 62 days for vehicle, cytarabine and NMS-P937 respectively) also in the established disease setting. Also in this case, our PLK 1 inhibitor was statistically different from cytarabine with p = 0.001.

**Figure 4:**
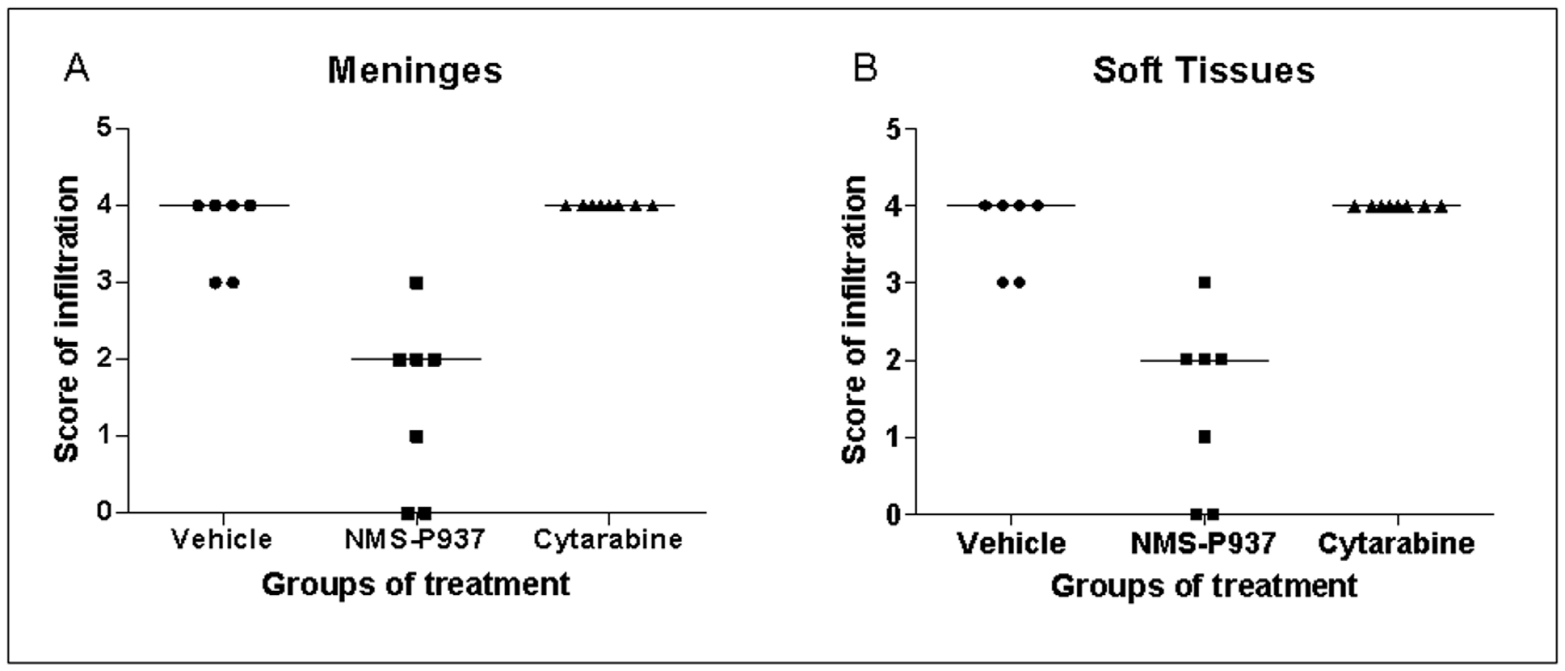
Leukaemic infiltration of meninges and soft tissues from mice treated with NMS-P937 and cytarabine following a therapeutic schedule. Mice were inoculated iv with 5×10^6^ AML-NS8 cells and treatments started at day 20, when leukaemic dissemination was present. Mice were treated with vehicle, NMS-P937 per os at 60 mg/kg bid per day over 2 days with 5 day rest and cytarabine at 75 mg/kg ip per day over 5 days with a 5 day rest, continuously until mice were moribund. Organs were collected for histological and immunohistochemical analysis. The graphs show the qualitative evaluation of leukaemic infiltration (expressed as score) in meninges (**A**) and soft tissues (**B**). Score: 0 = no infiltration, 1 = minimal, 2 = moderate, 3 = marked, 4 = severe infiltration.

Importantly, while % T/C (100× MST treated group/MST vehicle group) of cytarabine is comparable in the two different schedules (146% and 128% in the engraftment and established disease setting, respectively), NMS-P937 showed a better %T/C in the established disease (221% versus 175% in the engraftment setting). Histological analysis indicated that the PLK1 inhibitor significantly decreased (in 5 out of 7 cases) or abolished (in 2 out of 7 cases) leukaemic infiltration in the meninges and soft tissues compared to cytarabine or vehicle in a therapeutic protocol. These data were confirmed by anti-human HLA A,B,C immunohistochemical staining of the tissues (Figure 4).

### *In vitro* and *in vivo* Mechanism of Action of PLK1 Inhibitor NMS-P937 on AML-NS8 Cells

**Figure 5:**
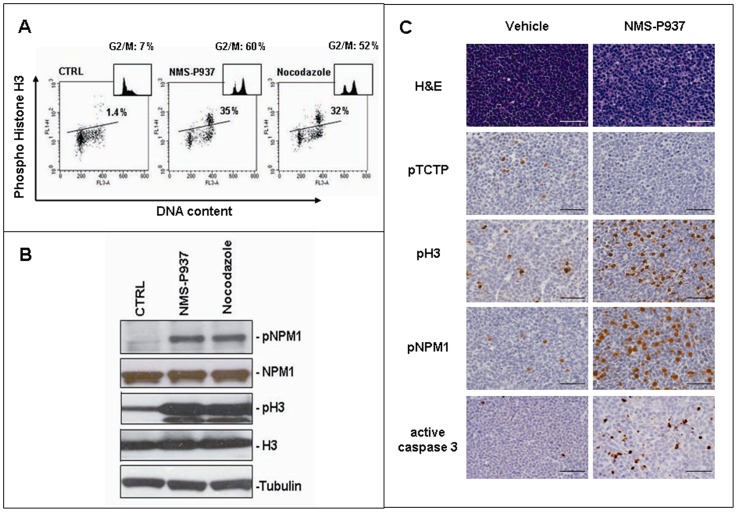
Mechanism of action of PLK1 inhibitor *in vitro* and *in vivo*. *In vitro* experiments: AML-NS8 cells were untreated (CTRL) or treated with 0.2 µM NMS-P937 for 24 hours or 0.75 ng/ml nocodazole for 16 hours. (**A**) biparametric analysis for phospho-Histone H3 and DNA content by flow cytometry. Inserts show the correspondent cell cycle profile and indicate the percentage of cells in G2/M phase. (**B**) Western blot analysis of total cell lysates for the following proteins: phospho-NPM1 (pNPM1), NPM1, phospho-Histone H3 (pH3), Histone H3 (H3). Tubulin protein expression was used as loading control. *In vivo* experiments: (**C**) Solid tumour masses from vehicle or NMS-P937 treated animals were collected, fixed and stained with H&E or antibodies against phospho-TCTP (pTCTP), phospho-Histone H3 (pH3), phospho-NPM1 (pNPM1) or active Caspase 3. Representative pictures are reported (Axio Scope Zeiss, magnification×400). Black/white bar, 50 µm.

PLK1 inhibitors have been shown to induce NPM1 and Histone H3 phosphorylation which are hallmarks of the mitotic block induced by the drug. Furthermore NMS-P937 inhibits phosphorylation of translational controlled tumour protein (TCTP), a direct target of PLK1 in solid tumours [28]. We therefore investigated the mechanism of action of NMS-P937 in AML-NS8 cells *in vitro*. As shown in Figure 5A, a 24 hour treatment induced an accumulation of cells in G2/M (60% vs 7% in control cells), similarly to what observed with the classical anti-mitotic drug Nocodazole (52%), to which corresponded a strong increase of phospho-Histone-H3 expression (1.4%, 35% and 32% in control, NMS-P937 and Nocodazole respectively). Western blot analysis of the same samples confirmed the modulation of phospho-Histone-H3 *in vitro* and revealed also the increased level of phospho-NPM1 expression compared to untreated cells (Figure 5B). We next studied the specific inhibition of PLK1 by NMS-P937 also *in vivo*, verifying if the compound was able to reach the extramedullary infiltrates. Vehicle and treated animals were monitored and when they showed manifestations of terminal disease they were given a boost of vehicle or NMS-P937 at 120 mg/kg and 6 hours later, at sacrifice, tumour masses and organs were collected. A clear modulation of PLK1 specific biomarkers was visible by immunohistochemistry. Indeed a complete inhibition of phospho-TCTP expression and induction of phospho-Histone H3 and phospho-NPM1 could be observed in skull and column masses from NMS-P937 treated animals (Figure 5C) and interestingly also in nearby meninges (Figure S3). These data demonstrate that our PLK1 inhibitor is able to modulate phosphorylation of specific substrates in extramedullary tumour infiltrates. Therefore, immunohistochemical staining for Caspase 3 activation revealed as NMS-P937 induces apoptosis as already reported for solid tumours [14] (Fig 5C).

## Discussion

Acute myeloid leukaemia is a heterogeneous disease, classifiable in different subtypes, which presents with highly variable and sometimes multiple cytogenetic, molecular and phenotypic abnormalities. Some of these aberrations correlate with response to therapy and survival. Despite significant progress in the treatment of AML, relapse and resistance are still common, calling for more effective therapies, whose development would require animal models recapitulating the patho-physiological features of these different disease entities [16]–[19], [22], [27]. In this report, we characterise in detail a new *in vivo* model of a primary disseminated AML, named AML-NS8, derived from the leukapheresis sample of a patient with a very aggressive CD56^+^ acute monoblastic leukaemia (M5a). In mice the cells engrafted well and expanded within the abdominal cavity with the production of both ascites and solid tumour masses. Expanded cells were very similar to the primary tumour in terms of morphology, immunophenotype and karyotype. Indeed Q-banding revealed that all leukaemic cells at the 5^th^ passage ip carried the double trisomy 8 and 6 already present in the leukapheresis sample at diagnosis. Besides, no other cytogenetic abnormalities or mutations were detected and the more refined genotype study by copy number analysis in SNP arrays demonstrated their genomic stability. In contrast, the same cells expanded *in vitro* for 5–6 weeks showed several additional chromosomal focal deletions and gains. These data support the view that, whereas the *in vitro* establishment of cell lines induces multiple genetic alterations that accumulate with time, *in vivo* expansion does not generally do so with such high frequency [17], [18], [20]. Thus the apparent identity of phenotype, genotype and general behaviour of the leukaemic cells at diagnosis and after *in vivo* passages suggests that these cells can indeed provide a predictive and reliable animal model of an aggressive AML.

Interestingly a biallelic loss of the p16^INK4^ gene was detected already at diagnosis in AML-NS8 cells. Biallelic mutations or deletions of this tumour suppressor gene is a common event in cancer [29] including leukemia [30] and is likely to play a role in the aggressiveness of the AML-NS8 tumour.

After *in vivo* stabilisation, expanded AML-NS8 cells were injected iv to induce disseminated disease which led to animal death within 28–30 days. The tumour dissemination was analysed in all haematopoietic and non haematopoietic organs using a variety of techniques including immunohistochemistry, flow cytometry and MRI. The data showed a clear infiltration of neoplastic cells in BM, lung, soft tissues and meninges in about 90% of mice and presence of solid masses in 25% of cases. Besides, small infiltrates of neoplastic cells were also present in spleen, blood, kidney, and liver. The infiltration of AML-NS8 cells within meninges of both column and skull and the documented presence of tumoural masses always associated with bones suggested that leukaemic cells in BM induced bone resorption, with consequent invasion of meninges on one side and muscles on the other, forming solid neoplasms.

Interestingly the overall distribution and localisation of neoplastic cells in extramedullary compartment and formation of solid masses as well as CNS and periodontal space involvement are common characteristics observed in patients AML-M5 [31], even if a direct correlation with the donor patient was not feasible due to his early death. Besides, CD56, expressed in 15–20% AML cases, is often associated with monocytic characteristics and with some chromosomal aberrations such as trisomy 8 [32]. It is also correlated, together with 11q23 abnormalities, with extramedullary and aggressive disease, CNS involvement, poor prognosis and early death [7]–[9], [11], [32]–[35]. CD56 is a neuronal cell adhesion molecule whose precise function and ligands are still unknown. Thus whether and how CD56 expressed by tumour cells may influence directly or indirectly the extramedullary localisation of leukaemic cells is an important question that still needs to be answered. There are indications that RUNX1 (AML1) isoforms can regulate CD56 expression [36], however it is still unclear how this may be linked to tumour aggressiveness. The AML-NS8 model described here may therefore be a useful tool to answer some of these questions.

AML-NS8 cells did not show genetic alterations therapeutically targetable so an alternative strategy could be represented by cell cycle inhibitors. PLK are a family of ser/thr kinases composed of 5 members, most of which are involved in cell cycle control. PLK1, that is the best studied of the family, phosphorylates cyclin B1, activates the cyclin B/Cdk1 complex and cooperates with Aurora kinases, thus controlling mitosis. It is also important for centrosome maturation, cohesion of sister chromatids and exit from mitosis [15], [26]. PLK1 is an attractive target in cancer, since it is overexpressed in a variety of solid tumours and in some leukaemias, most prominently in. AML [13], [37]. AML-NS8 cells were found to express this kinase and to respond to our PLK1 inhibitor NMS-P937 *in vitro* at nanomolar doses leading to G2/M arrest and increase of mitotic markers. A comparable activity was previously observed in several other AML cell lines, irrespective of FAB subtype [14]. Furthermore, in the *in vivo* disseminated AML-NS8 model, this novel oral targeted drug significantly prolonged survival compared to conventional therapies not only in the preemptive protocol, in which the compound was administered concomitantly with leukemic cells injection, but especially in the therapeutic protocol where the treatment started 20 days after tumour engraftment. With this schedule, better resembling the clinical condition of patients in treatment, our PLK1 inhibitor NMS-P937 had a stronger effect (MST = 62 days) compared to cytarabine (MST = 36 days) and vehicle (MST = 28 days) and produced a better %T/C than in the preemptive protocol (221% vs 175% respectively). Importantly in the therapeutic protocol, our PLK1 inhibitor significantly decreased or abolished leukaemic infiltration in meninges and soft tissues compared to cytarabine or vehicle. These promising results, even if obtained only in one case of aggressive AML, togheter with the efficacy of this inhibitor in other AML models [14] add further support to the possibility of targeting PLK1 in AML. Several PLK1 inhibitors have entered clinical studies in solid cancers and hematopoietic malignancies [14], [26]. Indeed a Phase I/II trial is ongoing with BI6727 in monotherapy or in combination with cytarabine in AML (clinicalTrial.gov NCT 00804856).

An important aspect in the evaluation of targeted therapies is the availability of reliable biomarkers to correlate the efficacy of a new drug to its mechanism of action and afterwards to translate in clinical practice. So, as suggested by Berg et al. [38], we put our efforts to define biomarkers reflecting treatment response in our model of AML. We could demonstrate inhibition of phosphorylation of pTCTP, a direct substrate of PLK1, and induction of phosphorylation of the mitotic markers Histone H3 and NPM1, other than increase of apoptosis, in meninges and extramedullary masses from NMS-P937 treated mice. This is of particular interest because it suggests that these molecular events are indeed the *in vivo* mechanism of action of the drug and it indicates that the AML-NS8 model is manageable for mechanistic studies. Finally, we could also show the possibility of following extramedullary infiltrations by MRI and this represents an additional tool for studying drug efficacy.

In summary, we demonstrate that this new mouse model of CD56^+^ AML provides a relevant system for integrating drug screening with biomarker evaluation. The efficacy of our PLK1 inhibitor NMS-P937 in this model supports its clinical development in leukaemias.

## Supporting Information

Figure S1 **Cytogenetic analysis of AML-NS8 samples.** (**A**) Bone marrow sample from patient at diagnosis. Cytogenetic analysis (Q-banding) showed the presence of 2 clones, one with trisomy 8 in 7 out of 22 metaphases (**A1**) and the other the double trisomy of chromosome 6 and 8 in 15 out of 22 metaphases (**A2**). (B) AML-NS8 cells expanded in mice. Cytogenetic analysis showed the double trisomy of chromosome 6 and 8. (**C**) FISH analysis. In vivo expanded cells cytocentrifuged and analyzed by FISH, confirmed the same evidence observed in B. The centromeres of chromosome 6 and chromosome 8 are stained in red and green respectively.
(TIF)

Figure S2 **Histopathological analysis and Magnetic Resonance Imaging (MRI) of bone structures.** SCID mice were inoculated iv with 5×10^6^ AML-NS8 cells and sacrificed upon manifestations of terminal disease. Column and skull were collected and fixed for histological analysis by H&E staining. The skull was also visualised by magnetic resonance imaging. (**A**) column transversal section (H&E) at ×25 magnification. Massive neoplastic cells infiltration of the muscle (ms), epidural space/meninges (es/m) and vertebral bone marrow (BM) was evident accompanied by areas of bone resorption (arrows). The neoplastic cells are stained blue. Black bar, 1 mm. (**B**) skull transversal section (H&E) at ×10 magnification. Deep neoplastic infiltration of epidural space/meninges (es/m). Arrow indicates macroscopic mass growing on the skull surface. Black bar, 1 mm. (**C**) T2-weighted MR image of the skull transversal section. A large area of meningeal neoplastic infiltration (indicated in yellow) is visible and surrounds the whole brain.
(TIF)

Figure S3 **Biomarker expression in meninges of vehicle or NMS-P937 treated animals.** Skull from vehicle or NMS-P937 treated animals were collected, fixed and paraffin embedded. Serial sections were stained with H&E or antibodies against phospho-TCTP (pTCTP), phospho-Histone H3 (pH3) or phospho-NPM1 (pNPM1). Severe infiltration by leukaemic cells was evident in the epidural space and meninges (es/m) under skull bone (b). As already seen in tumor masses, and as aspected due to its mechanism of action, NMS-P937 abolished the expression of its direct substrate pTCTP and increased mitotic markers also in meninges. Representative pictures at ×100 are reported. Inserts show high magnification at ×400. Black/white bar, 200 µm.
(TIF)
