# Supplementary figures and images for: The Polo-Like Kinase 1 (PLK1) Inhibitor NMS-P937 Is Effective in a New Model of Disseminated Primary CD56+ Acute Monoblastic Leukaemia
Source: PLoS One. 2013 Mar 8;8(3):e58424. doi: 10.1371/journal.pone.0058424 (PMC3592825; doi:10.1371/journal.pone.0058424)

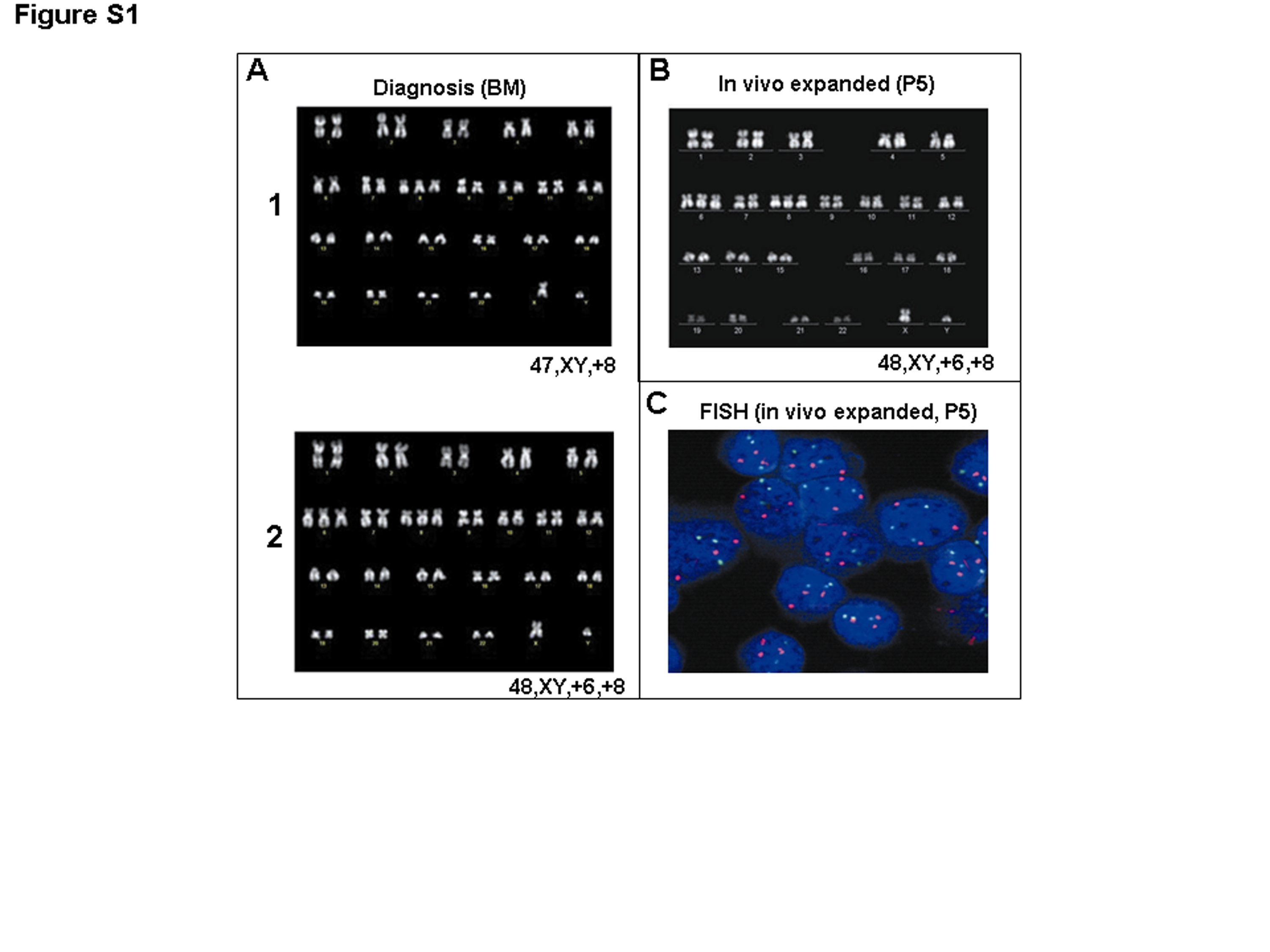

Supplement: Figure S1 — Cytogenetic analysis of AML-NS8 samples. (A) Bone marrow sample from patient at diagnosis. Cytogenetic analysis (Q-banding) showed the presence of 2 clones, one with trisomy 8 in 7 out of 22 metaphases (A1) and the other the double trisomy of chromosome 6 and 8 in 15 out of 22 metaphases (A2). (B) AML-NS8 cells expanded in mice. Cytogenetic analysis showed the double trisomy of chromosome 6 and 8. (C) FISH analysis. In vivo expanded cells cytocentrifuged and analyzed by FISH, confirmed the same evidence observed in B. The centromeres of chromosome 6 and chromosome 8 are stained in red and green respectively. (TIF) [file pone.0058424.s001.tif]

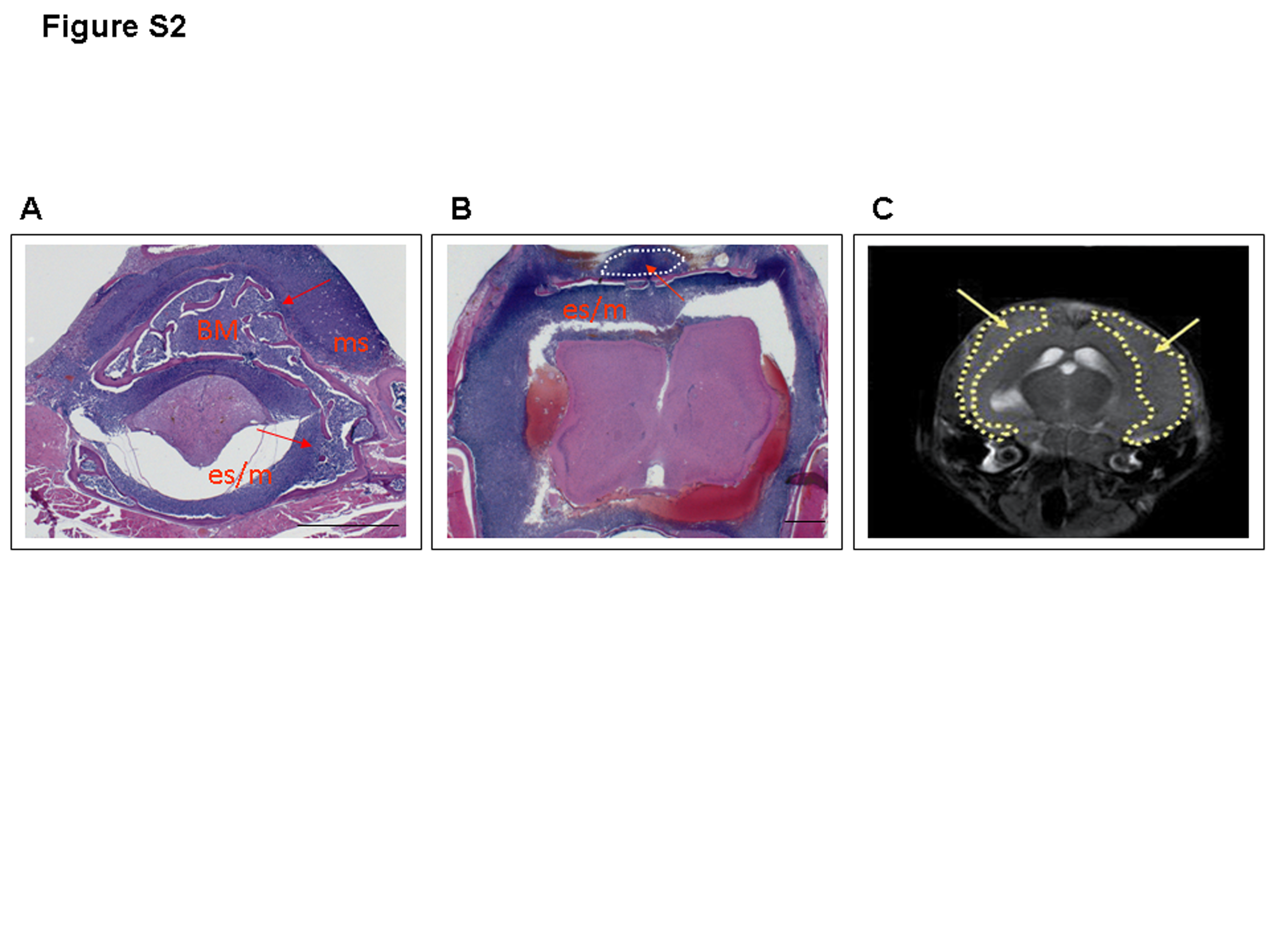

Supplement: Figure S2 — Histopathological analysis and Magnetic Resonance Imaging (MRI) of bone structures. SCID mice were inoculated iv with 5×10^6^ AML-NS8 cells and sacrificed upon manifestations of terminal disease. Column and skull were collected and fixed for histological analysis by H&E staining. The skull was also visualised by magnetic resonance imaging. (A) column transversal section (H&E) at ×25 magnification. Massive neoplastic cells infiltration of the muscle (ms), epidural space/meninges (es/m) and vertebral bone marrow (BM) was evident accompanied by areas of bone resorption (arrows). The neoplastic cells are stained blue. Black bar, 1 mm. (B) skull transversal section (H&E) at ×10 magnification. Deep neoplastic infiltration of epidural space/meninges (es/m). Arrow indicates macroscopic mass growing on the skull surface. Black bar, 1 mm. (C) T2-weighted MR image of the skull transversal section. A large area of meningeal neoplastic infiltration (indicated in yellow) is visible and surrounds the whole brain. (TIF) [file pone.0058424.s002.tif]

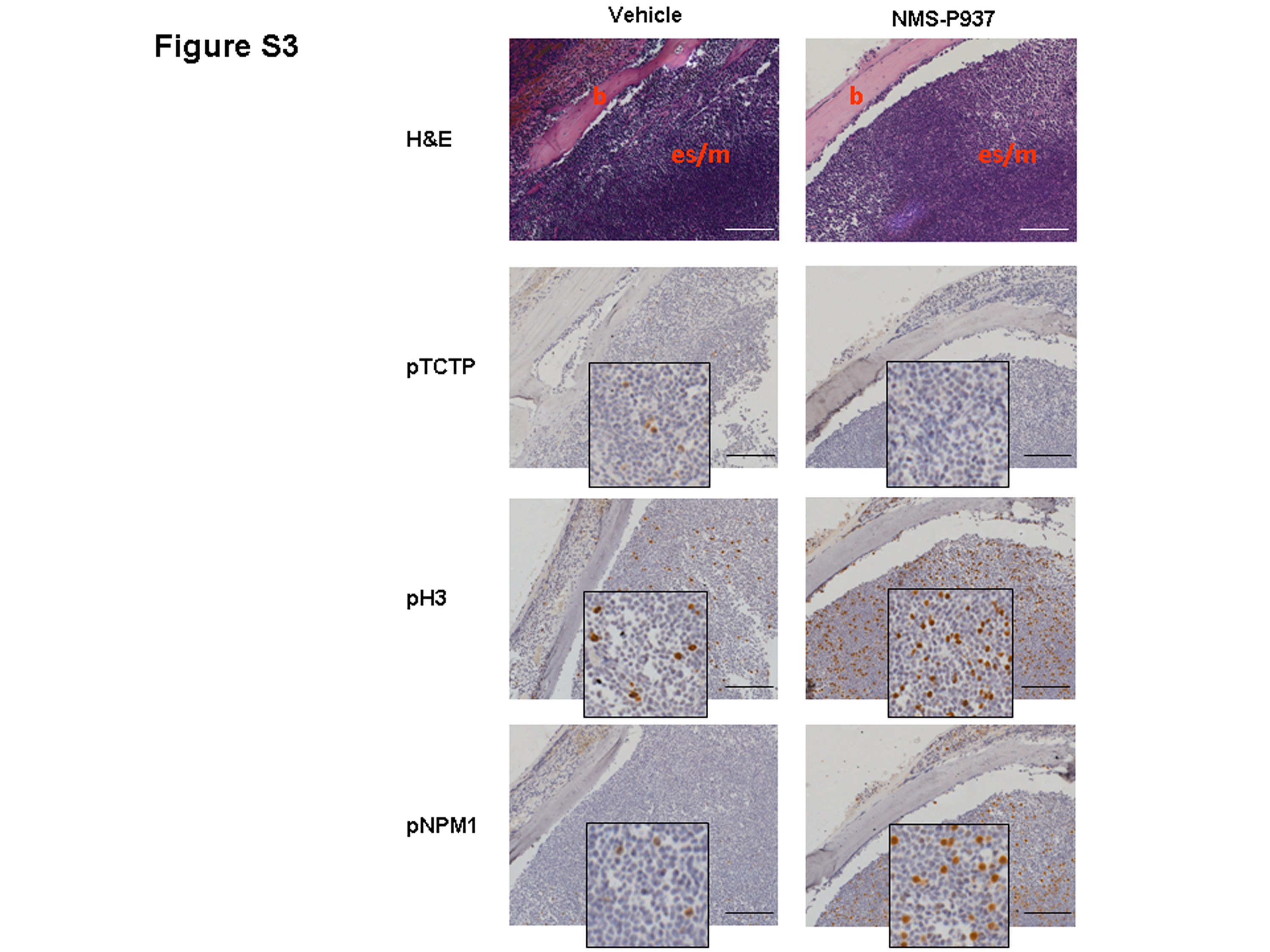

Supplement: Figure S3 — Biomarker expression in meninges of vehicle or NMS-P937 treated animals. Skull from vehicle or NMS-P937 treated animals were collected, fixed and paraffin embedded. Serial sections were stained with H&E or antibodies against phospho-TCTP (pTCTP), phospho-Histone H3 (pH3) or phospho-NPM1 (pNPM1). Severe infiltration by leukaemic cells was evident in the epidural space and meninges (es/m) under skull bone (b). As already seen in tumor masses, and as aspected due to its mechanism of action, NMS-P937 abolished the expression of its direct substrate pTCTP and increased mitotic markers also in meninges. Representative pictures at ×100 are reported. Inserts show high magnification at ×400. Black/white bar, 200 µm. (TIF) [file pone.0058424.s003.tif]
